# Salmagundi

**Published:** 1884-08

**Authors:** 


### Salmagundi.

EDITED BY E. G. BETTY, D.D.S.

Out of a total of 12,017 practicing dentists in this country, sixty-one are women.

"The patent acid drinks for summer, in too liberal quantities, are not only ruinous to the stomach, but also to the teeth."

To be kissed by the daughter and kicked out the back door by her father is what might be termed the "foot-and-mouth-disease."

By the way, what has become of all the "nascent" energy and latent force that made a couple of our exchanges so interesting a few months ago?

---

Dr. Will Taft's suggestion for smoothing the surface of oxy-phosphate fillings is a good one, and much cleaner than coating the surface with adhesive wax or paraffin.

---

We are not yet morally and scientifically certain just what the exciting causes of dental caries are. Will some one please rise and explain so that we may be relieved of the agony of suspense?

---

Dr. N. S. Hoff says that he uses the per oxide of hydrogen for obtunding sensitive dentine and with some satisfactory degree of success, especially in cases exhibiting no very marked degree of hyper-sensitiveness.

---

We do not believe in giving prominence to any nostrum of whatsoever kind or degree, but really, a half teaspoonful of Horsford's Acid Phosphate in a tumbler full of water makes a pleasant and refreshing drink during the hot weather.

---

Conventions seem to be the order of the day. Chicago has been taking a hand lately, and at the present writing (July 9th) its *Blaine* that there is fun ahead for us *all* in the *fall* when we don our war paint and begin a new session. New York may be heard from on some subjects, as Indiana already has.

---

Dr. Koch's experiments and researches upon cholera germs in Egypt last year do not seem to have any practical bearing so far, as genuine Asiatic cholera is now in the south of Europe threatening to devastate the entire continent, finally winding up with a whack at the cucumber eaters of the United States.

---

We would like to ask those who insist that carbolic acid is not an acid, but an alcohol, how they account for the union that takes place between carbolic acid and caustic soda to form what is known as Phenol Sodique, a body very different from either of its component parts, the usual result of chemical combination.

---

POWER (*Brit. Med. Journal*, Dec. 8, 1883) says it is now well known that dental irritation causes reflexly various eye affections, loss or failure of accomodation due to paralysis of the ciliary muscle being especially common, strabismus, ptosis, paralysis of the orbicularis, severe conjunctivitis, abscess of the cornea, amaurosis, and glaucoma.

---

WHAT is the reason that so few Eastern dentists contribute original articles for the Western journals? Can it be that they still regard us as "backwoodsmen," in spite of the fact that recent statistics of the timber area prove the term to be a misnomer? Maybe they are concentrating their energies to raise funds for the Bartholdi pedestal. In that case we might help the thing along by buying a good article occasionally.

---

DON'T forget that in packing a repair piece of rubber work, that if you will moisten the surface of the vulcanized rubber next the surface of the plate with chloroform, it will form an intimate union with the plate, thus tightly attaching itself to surfaces that can scarcely be so cut as to form a dove-tail or other device for retaining the added portion.

---

ANY of the readers of the "REGISTER" who are interested in forestry will find valuable information regarding "Forestal Experiment Stations" in Prof. Adolph Leue's paper on that subject, read at the Columbus meeting of the Ohio Forestry Association. The paper has been published in pamphlet form and any one desiring to read it can no doubt obtain a copy by addressing Prof. Adolph Leue, Eleventh District School, Cincinnati.

---

### A GOOD OPENING FOR A YOUNG MAN.

A Vienna dentist, who wishes to retire from business, advertises in the *Medizinische Zeitung* for "a young unmarried doctor who would like to succeed to a business of $5,000 a year, and have a very agreeable existence." No money is demanded for the business, and the agreeable existence is to be assured by marriage with the dentist's daughter, a handsome and well-educated girl of seventeen. A knowledge of dentistry is not an immediate requisite, as the advertiser will be pleased to show his son-in-law how to do plugging.—*Exchange.*

---

SANDUSKY, O., June 14, 1884.

ED. SALMAGUNDI: *Dear Sir*—I notice in "Salmagundi" a statement as to what Ohio ranks fifth in, viz.: number of dentists. Can you, and will you, give me the number of dentists in Ohio and Illinois, and oblige,

Truly yours,

E. J. WAYE.

The latest figures are, Ohio 650, Illinois 830.

---

A NEW base for artificial teeth, composed of 16 parts pure tin and 1 part bismuth, is proposed by Dr. N. W. Kingsley. It flows at the low temperature of four hundred degrees. The matrix he pours it in is composed of sea-sand and plaster, in which the teeth, as arranged, are imbedded. It is said to be very beautiful in appearance, and as little liable to discoloration as gold, and is easily repaired. If plain teeth are used, the gums and contour of celluloid or pink rubber is easily added.—*Items of Interest.*

---

EDITOR SALMAGUNDI:—In the May number of the *Ohio State Dental Journal* there appears a diarrhetic effusion by Prof. Nockemorf, Nockemup, or some similar cognomen, entitled "Dirty Birds," in which he treats of a specimen which he claims does not exactly show its nest that due respect to which it is entitled. Now, what we want to ask of the Professor is this, "If "Dirty Birds" make dirty nests, isn't it true on the other hand that dirty nests produce "Dirty Birds." Why don't somebody keep that nest clean?" W.

### THE ORIGIN OF THE WORD "CHARLATAN."

THE *Æsculapian* says: It is generally admitted that the word "charlatan" is derived from the Italian word *ciarlare*, to chatter, to prate. It appears that up to the sixteenth and seventeenth centuries the word was pronounced "chiarlatan." A German journal gives its etymology differently: A Paris physician, Latan, went through the town in a chariot containing his drugs, and in which he examined his patients. This caused the expression "*Voila le char de Latan*," which was subsequently corrupted into "charlatan."

---

TO ALL wearers of false teeth the news of the recent fashion set by a Chicago society lady will be extremely welcome. This lady has an entire set of false upper teeth, and she neither conceals the fact nor pretends that they are preferable to real teeth. She is also near-sighted and wears suspended to a hook on the northwest summit of her dress, a pair of neat eye-glasses, which she puts on whenever she wishes to look at anything. Some time ago it occurred to her that it would be the part of common sense to use her teeth only when she desired to talk or eat. Accordingly, she now carries them suspended by a cord around her neck. When she meets a friend she first puts on her eye-glasses and looks at him, and then puts in her teeth and indulges in conversation. Similarly, when she goes to dinner, she puts in her teeth as soon as the soup has disappeared and the fish is brought on. Being a leader of Chicago fashion, her example has been followed by other ladies, and at a Chicago opera quite a large proportion of the ladies of the audience wear their teeth gracefully suspended from the neck.—*N. Y. Times.*

---

### DR. ATKINSON'S LARGE BILL.

NEW YORK, July 4.—The $7,000 bill rendered by Dr. Atkinson, a well-known dentist of East Ninth street, to President Guzman Blanco, of Veuezuela, for four days' work, has not yet been paid, which may account for Dr. Atkinson's choleric receptions of inquiries made to-day as to the status of the case. When Mrs. Blanco, her two daughters, and her sister left for Venezuela on Saturday, they were thoroughly satisfied that their teeth were in admirable condition. The exorbitant bill charging $1,750 per day for the job, or $437.50 per patient per day, was not settled, a sum of money being left in the hands of a legal firm for arbitration. "It is a gross impertinence," said Dr. Atkinson to-day, "for the newspapers to be meddling in my business. It is morally wrong. If it were not for the good old Quaker stock, such as that from which I come, this country would not be fit for decent people to live in. The hellions seem to have things their own way. I won't say a word about my $7,000 bill. Its none of the public's business."—*Cin. Commercial Gazette.*

---

The thirty-ninth annual announcement of the Ohio College of Dental Surgery is out in fine form. Two features are promised, one of which is new, to wit:

"In addition to the superior advantages for practical training, which this Institution has heretofore afforded students, the Faculty will, the coming session, introduce the system of lighting by electricity, into the Infirmary Rooms of the College, and establish an evening clinical service;" and "The SUMMER SESSION for 1885 will begin the 1st day of April and continue until the 15th of September. The Summer Session is intended especially for practical instruction. The lectures given from time to time will treat mostly of practical subjects; and the large clinical business, of the Winter Session, which, by this arrangemnt, is carried over to the Summer Term, gives the student rare opportunities for applying principles in actual practice at the operating chair. Instruction in anatomy, physiology, histology, etc., will be mostly recitative, and the microscope will be largely used in the study of these and collateral branches."

The idea of performing clinical operations by electric light is at least novel, and if carried out will demonstrate whether artificial light can be made of practical service without wearing out the eyes of the operator. Summer sessions have been tried before with only partial success, and it is hoped that they will do much good by giving the student more time for practical work than the full course of lectures of the winter allow.

CINCINNATI, July 10, 1884.

EDITOR SALMAGUNDI:—Several years ago a dentist by the name of Ludwig traveled through the country, giving instructions in a process which he claimed when applied to oxychloride and phosphate fillings would prevent solubility, and render them more permanent. Of course we bit and paid his fee. Now while the effects were less than he claimed, yet we never regretted the payment of the money. This process consisted of rubbing heated soapstone points, prepared for the purpose, over the surface of the filling, making it smooth, and imparting a beautiful polish, and inciting the patient to exclaim: "Why, Doctor, how nice and smooth that seems—just like the tooth itself!" instead of "Doctor, how rough; can't you do something to make it feel better?" and to which you can only reply: "Never mind, the effect of mastication will bring it around all right." Now the satisfaction of having the patient pleased is compensation for the expenditure.

But, farther, we thought, if it is good for these fillings, why not for Hill's stopping, gutta percha, etc.; so we tried it, and found that after the materials were placed in the cavity, and the application of soapstone, the filling could be adapted to the walls as thoroughly as any filling in use, and not drawn away from them by the adherance to hot metallic instruments, as is the case where they are used: besides, a beautiful, smooth surface is secured. Now this is not new, as there are probably 12,000 dentists in the United States that know all about it, but there may be a few who have not heard about it as yet. This is for their benefit. However, instead of cursing Dr. Ludwig, as others have done, we have always been full of gratitude toward the "humbug" the doctor practiced upon us.

WM. TAFT.

---

A KANSAS paper says the female drug clerk in a temperance town is not a brilliant success. When you wink at her across a soda fountain she doesn't know whether to put a little Balm of Gilead in your soda or to hang her head and blush.

